# Suicide and all-cause mortality following routine hospital management of self-harm: Propensity score analysis using multicentre cohort data

**DOI:** 10.1371/journal.pone.0204670

**Published:** 2018-09-27

**Authors:** Sarah Steeg, Matthew Carr, Richard Emsley, Keith Hawton, Keith Waters, Harriet Bickley, Jennifer Ness, Galit Geulayov, Nav Kapur

**Affiliations:** 1 Centre for Mental Health and Safety, School of Health Sciences, University of Manchester, Manchester Academic Health Science Centre, Manchester, United Kingdom; 2 Biostatistics and Health Informatics, Institute of Psychiatry, King’s College London, London, United Kingdom; 3 Centre for Suicide Research, University of Oxford Department of Psychiatry, Warneford Hospital, Oxford, United Kingdom; 4 Centre for Self-Harm and Suicide Prevention Research, Derbyshire Healthcare NHS Foundation Trust, Derby, United Kingdom; 5 Greater Manchester Mental Health NHS Foundation Trust, Manchester, United Kingdom; University of Queensland, AUSTRALIA

## Abstract

**Background:**

Observational studies are suited to examining links between the routine hospital management of self-harm and future suicide and all-cause mortality due to their large scale. However, care must be taken when attempting to infer causal associations in non-experimental settings.

**Methods:**

Data from the Multicentre Study of Self-Harm in England were used to examine associations between four types of hospital management (specialist psychosocial assessment, general hospital admission, psychiatric outpatient referral and psychiatric admission) following self-harm and risks of suicide and all-cause mortality in the subsequent 12 months. Missing data were handled by multiple imputation and propensity score (PS) methods were used to address observed differences between patients at baseline. Unadjusted, PS stratified and PS matched risk ratios (RRs) were calculated.

**Results:**

The PSs balanced the majority of baseline differences between treatment groups. Unadjusted RRs showed that all four treatment types were associated with either increased risks or no change in risks of suicide and all-cause mortality within a year. None of the four types of hospital management were associated with lowered risks of suicide or all-cause mortality following propensity score stratification (psychosocial assessment and medical admission) and propensity score matching (psychiatric outpatient referral and psychiatric admission), though there was no longer an increased risk among people admitted to a psychiatric bed. Individuals who self-cut were at an increased risk of death from any cause following psychosocial assessment and medical admission. Medical admission appeared to be associated with reduced risk of suicide in individuals already receiving outpatient or GP treatment for a psychiatric disorder.

**Conclusions:**

More intensive forms of hospital management following self-harm appeared to be appropriately allocated to individuals with highest risks of suicide and all-cause mortality. PS adjustment appeared to attenuate only some of the observed increased risks, suggesting that either differences between treatment groups remained, or that some treatments had little impact on reducing subsequent suicide or all-cause mortality risk. These findings are in contrast to some previous studies that have suggested psychosocial assessment by a mental health specialist reduces risk of repeat self-harm. Future observational self-harm studies should consider increasing the number of potential confounding variables collected.

## Introduction

Preventing suicide is a global public health priority. Annually there are 11.4 suicides per 100,000 population and it is the leading cause of death for 15 to 29-year olds [1]. Using self-report measures, the World Health Organization estimates the global rate of suicide attempt is 400 per 100,000 [1]. This includes intentional self-injury and self-poisoning with or without fatal intent [1]. Throughout this study, we use the term self-harm to describe all intentional acts of self-poisoning and self-injury regardless of motivation [2]. This definition is used widely in research and practice in the UK and elsewhere [3]. While some countries distinguish between non-suicidal self-injury and suicide attempts, some evidence suggests that in practice there is often no clear division between the two categories [4]. Relatively few countries record national data on hospital attendances for self-harm. Estimates that do exist include self-harm rates per 100,000 population of 199 in Ireland [5], 150 in the US [6] and 315 in Sri Lanka (only including self-poisoning) [7]. In England, rates estimated from three urban centres were 362 for males and 441 for females [8]. There is a strong relationship between hospital treated self-harm and subsequent suicide. In one study, 0.5% of people were found to have died by suicide within a year of a hospital presentation to an Emergency Department (ED) [9]. In other studies, this was found to rise to 3.9% after five years [10] and to above 5% after nine years [11]. Individuals who have self-harmed are also at higher risk of premature death from other external causes and from natural causes [12]. Given the high risk of suicide after self-harm, particularly in the early aftermath, EDs are a key potential suicide prevention site [13]. They provide opportunities to assess, treat and arrange follow-up care for individuals while they are in hospital.

The treatment provided to individuals presenting to ED following self-harm can vary widely [14]. Recommended practice from England is for a mental health specialist to carry out a psychosocial assessment following each presentation of self-harm [15]. In addition, many patients will be admitted to a medical bed, particularly for physical treatment of the effects of their self-harm. Following discharge from hospital some will be referred for psychiatric outpatient care, and a small proportion will be admitted for psychiatric inpatient care.

Some studies have attempted to identify possible protective approaches to care following a hospital attendance. A randomised controlled trial (RCT) carried out in England provided therapeutic assessments to adolescents following an ED attendance for self-harm [16]. The intervention was associated with improvements in treatment adherence. A Japanese trial of assertive case management reported short term improvements in the incidence of self-harm repetition [17]. However, the scale of RCTs is usually too small to be able to detect differences in suicide and other early mortality outcomes.

Findings from observational studies, such as case-control and cohort studies, are better powered to measure suicide and other causes of mortality as outcomes. Some observational studies have suggested increased risks of mortality following hospital care. A case-control study carried out in Denmark reported increased risks of suicide in the year following emergency department treatment and psychiatric admission [18]. The increased risk is likely to be a result of patients at highest risk of suicide receiving the most intensive care. However, the possibility that psychiatric care, particularly more restrictive forms of treatment, could contribute to an increased risk has also been raised [19]. Other studies have reported stepwise increases in risk for individuals as the intensity of hospital care following self-harm (in terms of the urgency, frequency and site of care provided) increased [20, 21].

Inferring causality between treatment and an outcome is a major challenge with observational data. In randomised trials the risks of the outcome of interest following treatment would be expected to be equal if the treatment groups were switched (known as exchangeability) [22]. Subsequently, differences in risk can be assumed to be due to the treatment. In observational studies, the assumption of exchangeability is weakened to conditional exchangeability (the treatment groups are exchangeable conditional on measured characteristics but not unobserved characteristics).

Previous studies have employed propensity score methods to compare population-level treatments following self-harm [23, 24]. However, the possible effects of routine management have, to date, mainly been examined using traditional methods such as multivariable regression models by adjusting such models for measured confounding factors [21]. An important role of propensity score methods is to balance treatment groups on measured characteristics. The propensity score is defined as ‘the conditional probability of assignment to a particular treatment given a vector of observed covariates’ [25]. Propensity score methods offer a pragmatic approach to handle selection effect bias when using observational data. The observable differences between treated and untreated subjects can be specified and the degree to which they are subsequently balanced can be examined. This helps to interpret resulting effect estimates. Propensity score methods also allow comparisons to be made within specified populations, including within propensity score strata and between treated and untreated patients within a pre-specified distance in their propensity scores.

The average treatment effect (ATE) or the average treatment effect on the treated (ATT) can be estimated. If both treatment groups are represented across the full range of PSs, it is appropriate to estimate the ATE. If one or more of the treatment groups has a limited range of PSs, for example if a treatment was only given to individuals with high PSs and there were no treated individuals with lower PSs, an estimate of the ATT is more appropriate [26]. The ATE represents the treatment effect for the study population while the ATT represents the effect only in subjects within the population that could feasibly receive the treatment (according to the measured covariates). The ATT, therefore, only applies to the population that received the treatment rather than the study population.

The aim of the current study is to use propensity score methods to estimate the effects of four categories of care received after self-harm (specialist psychosocial assessment, general hospital admission, psychiatric outpatient referral and psychiatric admission) on suicide and all-cause mortality. We hypothesised that, following propensity score adjustment, individuals receiving each category of management would be at lower risk of suicide and all-cause mortality than those receiving no such care. In addition to overall associations, we were interested in differences between certain groups of individuals. There is evidence that effects of routine management of self-harm may differ by mental health care and self-harm history [27]. Effects of outpatient treatment following self-harm may be different according to gender and age group [21]. Finally, we examined differences between possible non-suicidal self-injury and suicide attempts (a binary classification used in some countries) [4]. We did this by comparing acts of self-cutting to those of self-poisoning and other types of self-injury such as asphyxiation and jumping from a height. These were proxy measures as information relating to suicidal intent was not available.

## Method

### Data sources

We used data from the Multicentre Study of Self-Harm in England [8], an observational cohort study on people who present to the ED having self-harmed. Each of the five hospitals participating in the Multicentre Study of Self-Harm in England has an established system for monitoring self-harm presentations [28]. Participating hospitals use a consistent definition of self-harm, which includes all intentional acts of self-harm regardless of motivation [2]. The cohort includes three cities in England, and data are combined from ED hospital records and assessments carried out by ED and/or mental health clinicians, and national mortality statistics. Data from the ED records and assessments included clinical variables such as method of self-harm (grouped into self-poisoning, self-cutting and other self-injury), psychiatric history and subsequent management. Data from the English Index of Multiple Deprivation (IMD), a measure of the relative deprivation of small areas in England [29], were also linked to the cohort. The full list of variables collected can be found in Table 1.

**Table 1:** Cohort characteristics by hospital management^1^ at baseline^2^.

Variable (% of treated with variable present)	All (31,725)	Specialist psychosocial assessment, 18,252/31,725 (57.5%)	No specialist psychosocial assessment, 13,473/31,725 (42.5%)	Medical admission, 15,738/25,270 (62.3%)	No medical admission, 9,532/25,270 (37.7%)	Psychiatric outpatient referral, 9,244/29,889 (30.9%)	No psychiatric outpatient referral, 20,645/29,889 (69.1%)	Psychiatric inpatient admission, 1,800/31,725 (5.7%)	No psychiatric inpatient admission, 29,925/31,725 (94.3%)
Female	58.0	58.9	56.8	59.3	56.2	58.4	58.3	51.9	58.4
Age 16 to 24	35.2	34.3	36.5	33.2	38.2	31.3	38.7	18.1	36.3
Age 25 to 44	45.1	44.6	45.8	44.2	46.0	45.8	44.6	46.3	45.1
Age 45 to 64	16.3	17.5	14.7	18.1	13.2	18.9	14.6	20.7	16.1
Age 65+	3.3	3.6	3.0	4.5	2.0	4.0	2.1	14.9	2.6
Self-poison	83.7	87.3	78.8	91.9	69.2	85.2	83.8	74.1	84.2
Self-cut	11.9	8.6	16.4	4.6	23.6	10.3	12.4	14.4	11.8
Other self-injury	4.4	4.1	4.9	3.5	7.1	4.5	3.8	11.5	4.0
Any current psychiatric treatment (including GP)	42.5	35.7	47.5	40.1	48.4	58.9	32.3	72.1	40.6
Any previous psychiatric treatment	54.7	51.2	59.4	54.8	60.1	67.2	47.2	79.2	53.4
*Previous self-harm*									
None	35.8	40.6	29.3	38.3	30.4	28.4	39.8	26.5	36.2
In the past year	30.9	29.6	32.8	29.8	36.0	39.7	25.9	45.9	30.2
More than 1 year ago	26.2	27.8	24.1	28.0	24.9	28.9	25.0	24.8	26.2
Time not known	7.0	2.0	13.8	3.9	8.7	2.9	9.3	2.8	7.3
Alcohol taken	59.0	55.6	63.6	59.2	58.8	56.5	61.4	37.8	59.9
*Problems precipitating self-harm*									
Relationship with partner	37.2	46.0	25.1	43.1	26.3	37.4	38.1	22.6	37.9
Relationship with family	19.0	26.0	9.6	24.9	13.4	24.7	16.9	15.8	19.3
Response to mental health symptoms	17.2	22.6	9.8	20.4	14.6	27.9	9.7	44.8	15.4
Work/study	13.5	19.1	5.9	18.1	8.2	18.1	11.6	11.6	13.6
Money	11.1	15.8	4.8	15.0	6.3	14.7	9.7	11.8	11.2
Housing	8.8	12.7	3.6	11.7	4.9	12.5	7.0	10.1	8.7
Physical health	8.1	11.4	3.6	10.6	5.3	10.3	6.9	11.3	7.9
Relationship with others	7.5	9.2	5.1	8.0	4.8	10.0	6.6	6.0	7.6
Bereavement	7.1	8.9	4.7	8.2	4.9	8.4	6.6	8.1	7.2
Drug misuse	5.4	7.3	2.8	6.6	4.2	7.9	4.3	5.7	5.4
Abuse ^3^	5.1	7.3	2.9	6.6	3.5	7.9	3.8	6.1	5.0
Mean IMD score (high = deprived)	31.6	28.2	36.1	25.7	32.9	26.2	34.4	24.7	32.0

^1^ Treatment categories are not mutually exclusive

^2^Pooled proportions for multiply imputed data

^3^ Current or past physical/sexual/emotional abuse

We included adults aged 16 years and over presenting between 2000 and 2010 for two of the centres, and from 2003 to 2010 for one centre, due to data availability. For the ‘admission to a medical ward’ treatment category, for one of the centres, data were only available from 2005. Each individual’s final episode of non-fatal self-harm, either before death, or within the study period, was included. The cohort included 31,725 individuals attending one of the study hospitals between 2000 and 2010. 42.1% (13,358) presented to Manchester, 30.5% (9,664) to Derby and 27.4% (8,703) to Oxford. Individuals were followed up until the end of 2012 and notifications of deaths were provided by the Data Linkage Service, part of the Health and Social Care Information Service [30]. The timing and causes of mortality, therefore, could be ascertained for individuals presenting to the ED with self-harm. Specifically, this information included date of death, cause of death, coroner’s verdict (where an inquest took place) and International Classification of Diseases (ICD) 10 codes [31].

The self-harm monitoring system in Oxford was approved by South Central–Berkshire National Research Ethics Service and Derbyshire Research Ethics Committee approved the study in Derby. Both were granted ethical approval to collect data for both local and

multicentre projects. South Manchester Research Ethics Committee reviewed the project in

Manchester. The project was deemed not to require approval as the monitoring is conducted as part of a clinical audit system. All centres have approval under Section 251 of the NHS Act (2006) to collect patient identifiable data without patient consent and Oxford, Derby and Manchester have consent to send patient details to the Data Linkage Service.

### Treatment categories

To enable us to examine effects of each management strategy separately, treatments were grouped into four main categories: psychosocial assessment by a mental health specialist, admission to a medical ward (including short stay medical assessment units), referral to psychiatric outpatient services (including community mental health teams, crisis resolution teams, drug and alcohol services and psychotherapy services) and admission to a psychiatric bed. Treatment categories were not mutually exclusive. For example, individuals could receive a specialist psychosocial assessment and then be referred to outpatient care.

Effects of each treatment category were estimated separately. The comparison groups were individuals not receiving the treatment of interest, combined into a single comparison group, regardless of other types of management allocated during their presentation. Propensity score methods (see ‘Statistical procedures’, below) helped to ensure that individuals in the comparison groups were similar to those in the treatment groups, in terms of their measured characteristics. For individuals referred for outpatient psychiatric care, those admitted to a psychiatric bed (n = 1,836) were excluded from the comparison group. Due to the acute and high-intensity nature of psychiatric admission, any effect is likely to obscure that of outpatient care. Furthermore, people who were admitted as a psychiatric inpatient would be likely to have received outpatient care after discharge [32].

### Outcome measures

The outcomes of interest were suicide and death from any cause within twelve months of the hospital presentation. Suicides were defined as deaths given ICD 10 codes of intentional self-harm (X60 to X84) or undetermined intent (Y10 to Y34) [31]. All-cause mortality included all death notifications from the Data Linkage Service.

Both suicide and all-cause mortality within the twelve months following an ED presentation for self-harm are relatively rare events [21, 33]. Assuming a suicide rate of 0.5% amongst one treatment group and 1% in another, 5065 individuals in each treatment group would provide 80% power to detect the difference between groups.

### Statistical procedures

#### Missing data

Missing data were handled in one of two ways. Variables with low proportions of missing data (<6%) included the four categories of hospital management, suicide and all-cause death, age, gender and IMD area-level deprivation score. These data were found to be missing at random and cases with any missing values were excluded, an acceptable approach when the percentage of missing data is small [34]. For variables with higher proportions of missing data (range 25% to 35%), and where subjects with complete data differed to those with missing data, multiple imputation using the chained equations approach [35, 36] was performed in Stata [37]. This approach was used for the following variables: current psychiatric treatment (any vs. none), previous psychiatric treatment (any vs. none), previous self-harm (no previous self-harm, self-harm in the past year, self-harm more than a year ago and previous self-harm but timing not known), alcohol use within 6 hours of the self-harm act, factors precipitating the self-harm (partner relationship, family relationship, relationship with others, work or studies, financial problems, housing problems, legal problems, physical health, mental health, drug misuse, bereavement and current or past physical, sexual or emotional abuse). The following additional factors were used to impute missing values: study hospital, age in years, gender (male or female), method of harm (self-poisoning, self-cutting and other self-injury), IMD area-level deprivation score, type of hospital care received (specialist psychosocial assessment, medical admission, referral to psychiatric outpatient care and psychiatric inpatient admission) and mortality outcome (alive, died by suicide or died from any cause).

#### Propensity score methods

We estimated a propensity score for each individual in the cohort. A propensity score represents the probability of treatment assignment based on observed characteristics [25]. We used a multivariable logistic regression with treatment allocation as the outcome (dependent) variable and the following predictor (independent) variables: age group, gender, method of harm, study site, use of alcohol within 6 hours of the self-harm, previous self-harm, previous and current psychiatric treatment, problems experienced around the time of the self-harm (see Table 1 for the full list) and IMD area-level deprivation score. The propensity score was estimated separately for each of the four categories of hospital management.

In order to determine the most appropriate propensity score approach, the ‘common support’ (in other words, the range of propensity scores that were represented by both treated and untreated individuals) was examined. There was good common support across all propensity score values for psychosocial assessment and medical admission (S2 and S3 Tables, S5 Table and S1 and S2 Figs). Therefore, the propensity scores were used to estimate an average treatment effect (ATE) for the study population by stratifying the risk ratios by propensity score quintile.

There was common support only for propensity scores up to 0.6 for individuals referred to outpatient mental health care; above this threshold there were few untreated individuals (n = 785) (S3 Fig). Similarly, for psychiatric admission, there were sufficient untreated individuals only in the propensity score range 0 to 0.2; above this threshold there were 149 untreated individuals (S5 Fig). For these two treatments, therefore, the propensity scores were used to match treated individuals to untreated individuals and the average treatment effect on the treated (ATT) was estimated. One-to one, greedy matching was performed [26]. Greedy matching optimises each match individually rather than considering the overall distance between pairs. This approach has been shown to work well in creating balanced groups [26]. Due to the limited numbers of untreated individuals for some of the propensity score values, replacement of untreated individuals (to the matching pool) was allowed. However, untreated individuals could not be used as a match more than five times [26].

#### Estimating risk ratios

Unadjusted risk ratios (RRs) between treatment groups within 12 months were calculated separately for suicide and all-cause mortality using log-binomial regression models in Stata (Version 13.1) [37]. For treatments where there were treated and untreated individuals across the range of propensity scores, the risk ratio estimates were stratified by propensity score quintile to estimate the ATE. Where matching was used, the RR within the matched group was calculated to estimate the ATT. Differences in estimates of treatment effect by subgroups were examined by including interaction terms within the propensity score stratified and matched models. Multiplicative interaction terms for sex, age, previous self-harm, current and past psychiatric treatment, method of harm, ethnic group and area-based deprivation level were included. Subgroup-specific risk ratios (RRs) were then estimated.

## Results

### Features of the cohort

18,252/31,725 (57.5%) individuals received an assessment by a mental health specialist, 15,738/25,270 (62.3%) were admitted to a medical bed, 9,244/29,889 (30.9%) were referred to outpatient psychiatric care and 1,800/31,725 (5.7%) were admitted to a psychiatric bed. Table 1 shows characteristics of individuals in each of the treatment groups. In general, allocation of more intensive forms of care (referral to outpatient psychiatric services and admission to a psychiatric bed) was associated with older age, existing and past psychiatric treatment, more recent history of self-harm and lower deprivation of individuals’ local area.

There were baseline differences between the treatment groups (Table 1). PS adjustment balanced all measured covariates between groups receiving specialist psychosocial assessment (defined as a standardised difference of <0.1) [38] (S1 Table). For the medical admission treatment group, imbalance remained in the method of harm used by individuals, though the degree of imbalance was reduced. All other covariates were balanced following PS adjustment (S4 Table). Matching balanced all the characteristics for psychiatric outpatient referral, with one exception: a greater proportion of patients were receiving current psychiatric treatment in the referred group (42.0%) than those not referred (37.0%) (S6 Table). For psychiatric admission, imbalance remained between treatment groups for proportions in current treatment (72.1% in the treated group vs. 57.9% in the untreated group), with previous self-harm (45.9% had harmed in the past year vs. 37.0% in the untreated group) and age group: fewer treated were aged 45–64 (20.8% vs. 30.2%) but more were aged over 65 (13.6% vs. 9.4%) in the untreated group (S8 Table).

### Suicide

217 (0.68%) individuals died by suicide in the 12 months following the self-harm episode. The rate of suicide was higher amongst individuals receiving each of the four categories of management, rising most sharply for individuals receiving a referral to outpatient psychiatric care (1.05%, 97 suicide deaths) and those admitted to a psychiatric bed (1.56%, 28 suicide deaths) (Table 2). Following adjustment for propensity score, the increased risks of suicide following specialist psychosocial assessment and medical admission remained (Table 2). After propensity score matching, referral to outpatient care continued to be associated with an increased risk of suicide with the magnitude of the association weakened, but there was no association between psychiatric admission and 12-month suicide risk (Table 2).

**Table 2:** Risk of 12-month suicide and all-cause mortality by hospital management.

	All (31,725)	Specialist psychosocial assessment (18,252), n (%)	Medical admission (15,738), n (%)	Psychiatric outpatient referral (9,244), n (%)	Psychiatric inpatient admission (1,800), n (%)
**Suicide**					
Events n/N (%)	217 (0.68)	139 (0.76)	180 (0.71)	97 (1.05)	28 (1.56)
Unadjusted RR (95% CI)		**1.31** (1.06 to 1.63)	**1.41** (1.14 to 1.75)	**2.38** (1.66 to 3.41)	**2.46** (1.77 to 3.42)
PS adjusted RR (95% CI)		1.48 (0.97 to 2.26)	**1.59** (1.06 to 2.40)	-	-
PS matched RR (95% CI)		-	-	**1.86** (1.17 to 2.94)	1.12 (0.65 to 1.92)
**All-cause mortality**					
Events n/N (%)	687 (2.17)	405 (2.22)	406 (2.58)	272 (2.94)	85 (4.72)
Unadjusted RR (95% CI)		1.06 (0.86 to 1.30)	**1.50** (1.30 to 1.73)	**1.85** (1.14 to 2.98)	**2.35** (2.09 to 2.63)
PS adjusted RR (95% CI)		0.97 (0.73 to 1.29)	**1.48** (1.16 to 1.90)	-	-
PS matched RR (95% CI)		-	-	**1.38** (1.09 to 1.75)	1.08 (0.79 to 1.47)

Bold text denotes statistically significant difference between groups.

### All-cause mortality

The proportion of individuals dying from any cause within 12 months of self-harm was 2.17% (687). There was no change in risk of death following specialist psychosocial assessment, prior to any PS adjustment. Individuals admitted to a medical bed, referred for outpatient psychiatric care or receiving psychiatric admission were at greater risk of death (see ‘Unadjusted RR’, Table 2). For specialist psychosocial assessment and medical admission, propensity score adjustment did not alter the risk ratios. Following propensity score matching, outpatient referral continued to be associated with an increase in risk of death, though to a smaller degree. The increased risk associated with psychiatric admission was no longer observed within the propensity score matched sample (see ‘PS matched RR’, Table 2).

### Subgroup interactions

#### Specialist psychosocial assessment

An interaction between the estimated effect of assessment and the method of self-harm used on risk of all-cause mortality was observed: individuals who cut themselves were at greater risk following assessment (RR 1.87, CI 1.19 to 2.96, p value for interaction = 0.004) compared to those who self-poisoned (RR 0.89, CI 0.63 to 1.27) or used a method of harm other than poisoning or cutting (RR 0.47, CI 0.17 to 1.30).

#### Medical admission

The increased risk of suicide following medical admission was even higher for females (RR 1.96, CI 1.02 to 3.75, p = 0.001) and those who harmed themselves using a method other than poisoning or cutting (RR 3.79, CI 1.59 to 9.03, p = 0.02). For individuals in treatment for a psychiatric disorder at the time of the self-harm, medical admission was associated with a decrease in the risk of 12-month suicide (RR 0.65, CI 0.43 to 0.97, p <0.001).

Individuals who used cutting to harm themselves were at higher risk of death from any cause (RR 2.01, CI 1.28 to 3.15, p = 0.02).

#### Psychiatric outpatient referral

The increased risk of all-cause mortality following referral to outpatient psychiatric care was more pronounced for adults aged between 45 and 64 (RR (2.05, CI 1.23 to 3.43, p = 0.03) than other age groups. For individuals with a history of self-harm in the past year, the increased risk of all-cause mortality associated with psychiatric outpatient referral was not seen (RR 0.87, CI 0.58 to 1.30, p = 0.02).

#### Psychiatric admission

The risk of all-cause death within 12 months of psychiatric admission was lower amongst those who had self-harmed within the past year (RR 0.67, CI 0.37 to 1.21, p = 0.02).

## Discussion

### Main results

Prior to adjustment, the receipt of any of the categories of hospital management was associated with either an increase or no change in risks of suicide and all-cause mortality. None of the four types of hospital management were associated with lowered risks of suicide or all-cause mortality following propensity score adjustment (either by stratification or matching methods), though there was no longer an increased risk among people admitted to a psychiatric bed.

Some interactions between subgroups and estimated treatment effects were found. Individuals who self-cut were at an increased risk of death from any cause following psychosocial assessment and medical admission. Medical admission appeared to be associated with reduced risk of suicide in individuals already receiving outpatient or GP treatment for a psychiatric disorder.

### Strengths and limitations

This study has made use of a large, population-level cohort, enabling suicide and mortality (typically outcomes with low event-rates) to be compared between treatment groups. The routine nature of the treatments means studying their effects in an experimental setting is difficult. The use of propensity score methods to address observed sources of selection bias is a rigorous approach in this setting. However, the approach does have limitations. There is likely to be remaining imbalance in unobserved characteristics between treatment groups. Any observed links between treatments and outcomes cannot be assumed to be causal and should be interpreted with this caveat. The associations may be due, instead, to unmeasured baseline differences between individuals who received different treatment. There were also some measured differences between treatment groups after PS adjustment. Although the numbers of individuals receiving most treatment types were adequately powered to detect differences in suicide rates between treatment groups, the group receiving psychiatric inpatient admission was relatively small. This limited the statistical power for this estimate of treatment effect, particularly for detecting smaller differences.

This study examined broad categories of hospital management. For example, a referral to outpatient mental health care could involve community mental health care, crisis resolution teams, drug and alcohol teams or psychological therapy. It was not possible to examine effects of specific aspects of treatment. Furthermore it was not possible to ascertain if individuals received the offers of follow-up care as intended, and if they did, the length of treatment received. Future study designs could focus on linking hospital data to outpatient and primary care data to examine these questions in greater detail.

### Clinical and research implications

In unadjusted analyses, most aspects of hospital care following self-harm were associated with increased risks of suicide and all-cause mortality. This is in line with research from other countries, and suggests services are appropriately identifying treatment needs. Following propensity score methods to address observed confounding factors, risks following specialist psychosocial assessment and psychiatric admission were attenuated, suggesting some of the selection bias was addressed by the PS methods. It is likely that there were important unmeasured confounding factors that, had we been able to account for, may have altered our results.

The increased risks of suicide and all-cause mortality observed following referral to outpatient care could reflect incomplete PS balancing (i.e. residual observed differences between treatment groups), residual unobserved confounding or risks associated with treatment. The findings may reflect shortcomings in the aftercare provided for people referred to outpatient mental health services, the lack of evidence for effective interventions or they may result from incomplete adjustment for confounding factors.

The results from this study differed from an observational study conducted using Japanese general hospital data [39]. Patients presenting with drug overdose were compared between two groups: those who received psychiatric intervention (consisting of assessment only or assessment and psychotherapy) and those discharged without such intervention. Rates of readmission, following propensity score matching, were lower for those receiving the intervention. However, this study did not measure suicide and mortality outcomes, which may not be related to the intervention in the same ways. Two Danish studies compared risks of repeated self-harm, suicide and all-cause mortality in individuals taking part in psychosocial therapy following self-harm [23, 40]. The therapy was associated with reductions in all three outcomes. However, the intervention was more structured and consistent; the ‘suicide prevention clinics’ initiated contact and offered eight to ten sessions of therapy. The ‘outpatient’ intervention in the present study was more varied and could include relatively high-intensity interventions such as crisis resolution home treatment (CRHT), referrals to drug and alcohol community treatment teams, outpatient appointment with a psychiatrist, community mental health care or a combination of referrals. A Canadian population-based study examined the timing of follow-up in individuals treated for self-harm in the ED [41]. Those who received timely follow-up (within 30 days) had more chronic and severe mental illness. After adjusting for this and other baseline characteristics, no associations between specialist psychiatric or general practitioner contact and reduced risk of repeat self-harm were found.

A recent systematic review of RCTs of psychosocial treatments for adults who had self-harmed found evidence (of moderate quality) that cognitive-behavioural-based psychotherapy could help prevent repeat self-harm [42]. However, studies included in this review did not all record suicide outcomes. Trials of brief intervention in the Western Pacific areas of the World Health Organization have used information sessions and regular, brief follow-up contact. Meta-analysis of these trials suggested the receipt of the intervention was associated with a significant reduction in suicide [43].

Increased risks following medical admission and referral to psychiatric outpatient care were not seen for individuals with a history of self-harm and psychiatric treatment. The reduced risk of suicide following medical admission for these individuals could reflect benefits of spending time in hospital with more time for existing follow-up support to be arranged. The self-harm presentation may have helped to trigger an increase in treatment intensity, helping to re-establish contact with sources of care. This could have provided some protection against future self-harm and mortality for the year ahead, which was not received by those who were not known to services beforehand. However, the level of care received before the referral following self-harm, in terms of treatment intensity and setting (e.g. outpatient or primary care), was not known for individuals in this study.

Expanding the scope of the variables included in self-harm cohorts may help to address potential hidden confounding when comparing patient outcomes. A systematic review of risk factors for repeat self-harm found that the presence of depressive symptoms was a key risk factor [15]. However, the presence of depression was not measured in this study. A systematic review of risk factors for suicide following self-harm identified four factors that were independently significant, after adjusting for potential confounders [44]. While three of these were available for the present study (male gender, previous self-harm and physical health problems), one (suicidal intent) was not. A validated measure of suicidal intent, such as the SIS scale [45] could be an important potential confounder that was missing from this study.

### Conclusions

Most aspects of hospital management following self-harm were allocated to those with subsequent increased risks of suicide and all-cause mortality, suggesting individuals most at risk were receiving them. While some of these risks were attenuated following PS methods, there were no aspects of hospital management that were associated with lower risks of subsequent death. Results from this study may reflect incomplete adjustment for confounding effects. More information on potential confounding factors may alter our estimates treatment effect. In addition, more detailed information about the specific approach used as part of an individual’s routine clinical care following self-harm may help to elucidate the relationship between hospital management and mortality risks. Future studies may be able to build further on the evidence presented here by obtaining more detailed information about the content of the routine clinical management following self-harm. Some of these factors are routinely assessed as part of the specialist psychosocial assessment, increasing the feasibility of including them in future cohort studies.

## Supporting information

S1 Table (DOCX)

S2 Table (DOCX)

S3 Table (DOCX)

S4 Table (DOCX)

S5 Table (DOCX)

S6 Table (DOCX)

S7 Table (DOCX)

S8 Table (DOCX)

S9 Table (DOCX)

S1 Fig (DOCX)

S2 Fig (DOCX)

S3 Fig (DOCX)

S4 Fig (DOCX)

S5 Fig (DOCX)

S6 Fig (DOCX)
