# Supplementary material for: Suicide and all-cause mortality following routine hospital management of self-harm: Propensity score analysis using multicentre cohort data
Source: PLoS One. 2018 Sep 27;13(9):e0204670. doi: 10.1371/journal.pone.0204670 (PMC6161837; doi:10.1371/journal.pone.0204670)
Supplement: S1 Table — (DOCX) [file pone.0204670.s001.docx]

**S1 Table**: Specialist psychosocial assessment: Baseline vs. PS stratified characteristics of the imputed sample^1^ (N=31,725)

| Subgroup | Baseline untreated, % | Baseline treated, % | Standardised difference | Stratified untreated, % | Stratified treated, % | Standardised difference |
| --- | --- | --- | --- | --- | --- | --- |
| Total | 42.5 (13,473) | 57.5 (18,252) |  |  |  |  |
| Male | 43.2 | 41.1 | -0.04 | 41.1 | 41.1 | 0.001 |
| Female | 56.8 | 58.9 | 0.04 | 58.9 | 58.9 | -0.001 |
|  |  |  |  |  |  |  |
| Age 16 to 24 | 36.5 | 34.3 | -0.05 | 34.2 | 34.3 | 0.001 |
| Age 25 to 44 | 45.8 | 44.6 | -0.03 | 44.7 | 44.6 | -0.002 |
| Age 45 to 64 | 14.7 | 17.5 | 0.08 | 16.7 | 17.5 | 0.02 |
| Age 65+ | 3.0 | 3.6 | 0.04 | 4.4 | 3.6 | -0.04 |
|  |  |  |  |  |  |  |
| Self-poison | 78.8 | 87.3 | 0.23 | 86.5 | 87.3 | 0.02 |
| Self-cut | 16.4 | 8.6 | -0.24 | 11.0 | 8.6 | -0.08 |
| Other self-injury | 4.9 | 4.1 | -0.04 | 2.5 | 4.1 | 0.09 |
|  |  |  |  |  |  |  |
| Any previous psychiatric treatment | 59.4 | 51.2 | -0.18 | 52.8 | 51.2 | -0.03 |
|  |  |  |  |  |  |  |
| *Previous self-harm* |  |  |  |  |  |  |
| None | 29.3 | 40.6 | 0.24 | 41.6 | 40.6 | -0.02 |
| In the past year | 32.8 | 29.6 | -0.08 | 30.1 | 29.5 | -0.01 |
| More than 1 year ago | 24.1 | 27.8 | 0.10 | 19.9 | 27.9 | 0.19 |
| Time not known | 13.8 | 2.0 | -0.45 | 8.4 | 2.0 | -0.29 |
|  |  |  |  |  |  |  |
| Alcohol taken | 63.6 | 55.6 | -0.14 | 55.3 | 55.8 | 0.01 |
|  |  |  |  |  |  |  |
| *Problems precipitating self-harm* |  |  |  |  |  |  |
| Relationship with partner | 25.1 | 46.0 | 0.45 | 43.3 | 46.0 | 0.05 |
| Relationship with family | 9.6 | 26.0 | 0.44 | 25.2 | 26.0 | 0.02 |
| Relationship with others | 5.1 | 9.2 | 0.15 | 8.2 | 9.2 | 0.04 |
| Work/study | 5.9 | 19.1 | 0.41 | 18.5 | 19.2 | 0.02 |
| Money | 4.8 | 15.8 | 0.36 | 15.7 | 15.8 | 0.003 |
| Housing | 3.6 | 12.7 | 0.34 | 11.9 | 12.6 | 0.02 |
| Substance misuse | 2.8 | 7.3 | 0.20 | 6.7 | 7.3 | 0.02 |
| Physical health | 3.8 | 11.4 | 0.30 | 10.8 | 11.5 | 0.02 |
| Response to mental health symptoms | 9.8 | 22.6 | 0.36 | 21.2 | 22.6 | 0.04 |
| Bereavement | 4.7 | 8.9 | 0.16 | 8.4 | 8.9 | 0.02 |
| Abuse | 2.0 | 7.3 | 0.26 | 6.9 | 7.4 | 0.02 |
|  |  |  |  |  |  |  |
| Mean IMD score (high = deprived) | 36.1 | 28.2 | -0.39 | 26.9 | 28.2 | 0.07 |
|  |  |  |  |  |  |  |

*^1^Pooled proportions for multiply imputed data*
