# Supplementary material for: Suicide and all-cause mortality following routine hospital management of self-harm: Propensity score analysis using multicentre cohort data
Source: PLoS One. 2018 Sep 27;13(9):e0204670. doi: 10.1371/journal.pone.0204670 (PMC6161837; doi:10.1371/journal.pone.0204670)
Supplement: S2 Table — (DOCX) [file pone.0204670.s002.docx]

**S2 Table:** Specialist psychosocial assessment: Treated and untreated individuals by propensity score quintile

| Propensity score quintile | Not assessed (N) % | Assessed (N) % |
| --- | --- | --- |
| 1 | 5,085 (79.8) | 1,291 (20.2) |
| 2 | 4,378 (68.8) | 1,990 (31.3) |
| 3 | 2,817 (44.3) | 3,539 (55.7) |
| 4 | 935 (14.8) | 5,380 (85.2) |
| 5 | 258 (4.1) | 6,052 (95.9) |
| Total | 13,473 (42.5) | 18,252 (57.5) |
