# Supplementary material for: Suicide and all-cause mortality following routine hospital management of self-harm: Propensity score analysis using multicentre cohort data
Source: PLoS One. 2018 Sep 27;13(9):e0204670. doi: 10.1371/journal.pone.0204670 (PMC6161837; doi:10.1371/journal.pone.0204670)
Supplement: S3 Table — (DOCX) [file pone.0204670.s003.docx]

**S3 Table:** Specialist psychosocial assessment: Mean and range of propensity score by treatment group

|  | **No. of observations** | **Mean PS** | **Minimum PS** | **Maximum PS** |
| --- | --- | --- | --- | --- |
| Untreated | 13,473 | .3984401 | .0495249 | .9945188 |
| Treated | 18,252 | .7058852 | .0436339 | .9998305 |
| Total | 31725 | .5753191 | .0436339 | .9998305 |

There was common support of treated and untreated individuals across the range of propensity scores, suggesting the average treatment effect could be estimated for the whole cohort.
