# Supplementary material for: Suicide and all-cause mortality following routine hospital management of self-harm: Propensity score analysis using multicentre cohort data
Source: PLoS One. 2018 Sep 27;13(9):e0204670. doi: 10.1371/journal.pone.0204670 (PMC6161837; doi:10.1371/journal.pone.0204670)
Supplement: S4 Table — (DOCX) [file pone.0204670.s004.docx]

**S4 Table**: Medical admission: Baseline vs. PS stratified covariate balance of PS factors by medical admission, imputed data (N=25,270^1^)

| Subgroup | Baseline untreated, % | Baseline treated, % | Standardised difference | Stratified untreated, % | Stratified treated, % | Standardised difference |
| --- | --- | --- | --- | --- | --- | --- |
| Total | 37.7 (9,532) | 62.3 (15,738) |  |  |  |  |
| Male | 43.8 | 40.7 | -0.06 | 40.8 | 40.9 | 0.002 |
| Female | 56.2 | 59.3 | 0.06 | 59.2 | 59.1 | -0.002 |
|  |  |  |  |  |  |  |
| Age 16 to 24 | 38.2 | 33.2 | -0.10 | 31.0 | 33.5 | 0.05 |
| Age 25 to 44 | 46.0 | 44.2 | -0.04 | 46.8 | 44.7 | -0.04 |
| Age 45 to 64 | 13.8 | 18.1 | 0.12 | 18.2 | 17.4 | -0.02 |
| Age 65+ | 2.0 | 4.5 | 0.14 | 4.0 | 4.4 | 0.02 |
|  |  |  |  |  |  |  |
| Self-poison | 69.2 | 91.9 | 0.60 | 87.9 | 92.2 | 0.14 |
| Self-cut | 23.6 | 4.6 | -0.57 | 12.4 | 4.4 | -0.29 |
| Other self-injury | 7.1 | 3.5 | -0.16 | 0.3 | 3.4 | 0.31 |
|  |  |  |  |  |  |  |
| Any previous psychiatric treatment | 60.1 | 54.8 | -0.11 | 53.9 | 53.9 | -0.01 |
|  |  |  |  |  |  |  |
| Any current psychiatric treatment (including GP) | 48.4 | 40.1 | -0.17 | 40.7 | 39.4 | -0.03 |
|  |  |  |  |  |  |  |
| *Previous self-harm* |  |  |  |  |  |  |
| None | 30.4 | 38.3 | 0.17 | 38.9 | 39.5 | 0.01 |
| In the past year | 36.0 | 29.8 | -0.13 | 30.7 | 28.9 | -0.04 |
| More than 1 year ago | 24.9 | 28.0 | 0.07 | 25.4 | 27.5 | 0.05 |
| Time not known | 8.7 | 3.9 | -0.20 | 5.1 | 4.1 | -0.05 |
|  |  |  |  |  |  |  |
| Alcohol taken | 58.8 | 59.2 | 0.01 | 57.6 | 59.0 | 0.03 |
|  |  |  |  |  |  |  |
| *Problems precipitating self-harm* | |  |  |  |  |  |
| Relationship with partner | 26.3 | 42.9 | 0.35 | 42.2 | 44.0 | 0.04 |
| Relationship with family | 13.4 | 24.9 | 0.30 | 24.5 | 25.7 | 0.03 |
| Relationship with others | 4.8 | 8.0 | 0.13 | 6.7 | 7.9 | 0.04 |
| Work/study | 8.2 | 18.2 | 0.30 | 16.6 | 18.7 | 0.05 |
| Money | 6.3 | 15.2 | 0.29 | 14.0 | 15.4 | 0.04 |
| Housing | 4.9 | 11.5 | 0.24 | 10.3 | 11.7 | 0.04 |
| Substance misuse | 4.2 | 6.7 | 0.11 | 6.0 | 6.8 | 0.04 |
| Physical health | 5.3 | 10.6 | 0.20 | 10.7 | 10.8 | 0.01 |
| Response to mental health symptoms | 14.6 | 20.3 | 0.15 | 18.5 | 19.5 | 0.03 |
| Bereavement | 4.9 | 8.3 | 0.14 | 7.2 | 8.1 | 0.03 |
| Abuse | 3.5 | 6.6 | 0.14 | 5.8 | 6.3 | 0.02 |
|  |  |  |  |  |  |  |
| Mean IMD score (high = deprived) | 32.9 | 25.7 | -0.38 | 24.1 | 24.2 | 0.04 |
|  |  |  |  |  |  |  |

*^1^Pooled proportions for multiply imputed data*

17 variables were not balanced prior to PS stratification. Most were balanced after PS stratification but imbalance remained in method of harm, with a higher proportion of individuals admitted to a medical bed using self-poisoning as the method of harm, though the degree of imbalance was smaller.
