# Supplementary material for: Suicide and all-cause mortality following routine hospital management of self-harm: Propensity score analysis using multicentre cohort data
Source: PLoS One. 2018 Sep 27;13(9):e0204670. doi: 10.1371/journal.pone.0204670 (PMC6161837; doi:10.1371/journal.pone.0204670)
Supplement: S5 Table — (DOCX) [file pone.0204670.s005.docx]

**S5 Table:** General hospital admission: Mean and range of propensity score by treatment group

|  | **No. of observations** | **Mean PS** | **Minimum PS** | **Maximum PS** |
| --- | --- | --- | --- | --- |
| Untreated | 9532 | .5159744 | .0465172 | .9743912 |
| Treated | 15738 | .6874909 | .0592172 | .9894127 |
| Total | 25270 | .6123568 | .0465172 | .9894127 |

There was, broadly, good common support across propensity score quintiles. This suggested it was appropriate to estimate an average treatment effect for the population.
