# Supplementary material for: Suicide and all-cause mortality following routine hospital management of self-harm: Propensity score analysis using multicentre cohort data
Source: PLoS One. 2018 Sep 27;13(9):e0204670. doi: 10.1371/journal.pone.0204670 (PMC6161837; doi:10.1371/journal.pone.0204670)
Supplement: S6 Table — (DOCX) [file pone.0204670.s006.docx]

**S6 Table**: Outpatient referral: Baseline vs. PS matched covariate balance of PS factors, imputed data (N=29,889^1^)

| Subgroup | Baseline untreated, % | Baseline treated, % | Standardised difference | Matched untreated, % | Matched treated, % | Standardised difference |
| --- | --- | --- | --- | --- | --- | --- |
| Total | 69.1 (20,645) | 30.9 (9,244) |  | 50.0  (6,091) | 50.0  (6,091) |  |
| Male | 41.7 | 41.6 |  | 41.8 | 42.7 |  |
| Female | 58.3 | 58.4 | 0.001 | 58.2 | 57.3 | -0.02 |
|  |  |  |  |  |  |  |
| Age 16 to 24 | 38.7 | 31.3 | -0.17 | 34.3 | 35.3 | 0.02 |
| Age 25 to 44 | 44.6 | 45.8 | 0.03 | 43.2 | 44.6 | 0.03 |
| Age 45 to 64 | 14.6 | 18.9 | 0.13 | 18.5 | 16.5 | -0.05 |
| Age 65+ | 2.1 | 4.0 | 0.11 | 3.9 | 3.5 | -0.02 |
|  |  |  |  |  |  |  |
| Self-poison | 83.8 | 85.2 | 0.04 | 84.3 | 84.6 | 0.02 |
| Self-cut | 12.4 | 10.3 | -0.07 | 11.5 | 10.6 | 0.04 |
| Other self-injury | 3.8 | 4.5 | 0.04 | 4.3 | 4.8 | 0.02 |
|  |  |  |  |  |  |  |
| Any previous psychiatric treatment | 47.2 | 67.2 | 0.41 | 55.4 | 56.3 | 0.02 |
|  |  |  |  |  |  |  |
| Any current psychiatric treatment (including GP) | 32.3 | 58.9 | 0.55 | 37.0 | 42.0 | 0.10 |
|  |  |  |  |  |  |  |
| *Previous self-harm* |  |  |  |  |  |  |
| None | 39.8 | 28.4 | -0.24 | 37.6 | 33.4 | -0.09 |
| In the past year | 25.9 | 39.7 | 0.30 | 29.9 | 34.5 | 0.09 |
| More than 1 year ago | 25.0 | 28.9 | 0.09 | 25.2 | 28.9 | 0.08 |
| Time not known | 9.3 | 2.9 | -0.27 | 7.4 | 3.2 | -0.19 |
|  |  |  |  |  |  |  |
| Alcohol taken | 61.4 | 56.5 | -0.10 | 59.3 | 59.6 | 0.01 |
|  |  |  |  |  |  |  |
| *Problems precipitating self-harm* | |  |  |  |  |  |
| Relationship with partner | 38.1 | 37.4 | -0.02 | 40.5 | 39.3 | -0.02 |
| Relationship with family | 16.9 | 24.7 | 0.19 | 24.0 | 22.1 | -0.04 |
| Relationship with others | 6.6 | 10.0 | 0.12 | 9.2 | 8.5 | -0.03 |
| Work/study | 11.6 | 18.1 | 0.18 | 19.3 | 17.0 | -0.06 |
| Money | 9.7 | 14.7 | 0.16 | 15.7 | 13.4 | -0.07 |
| Housing | 7.0 | 12.5 | 0.19 | 12.7 | 10.2 | -0.08 |
| Substance misuse | 4.3 | 7.9 | 0.15 | 7.5 | 6.8 | -0.03 |
| Physical health | 6.9 | 10.3 | 0.12 | 10.3 | 9.4 | -0.03 |
| Response to mental health symptoms | 9.7 | 27.9 | 0.48 | 19.5 | 16.5 | -0.08 |
| Bereavement | 6.6 | 8.4 | 0.07 | 8.5 | 7.5 | -0.04 |
| Abuse | 3.8 | 7.9 | 0.18 | 7.5 | 5.7 | -0.07 |
|  |  |  |  |  |  |  |
| Mean IMD score (high = deprived) | 34.4 | 26.2 | -0.39 | 27.4 | 28.9 | 0.06 |
|  |  |  |  |  |  |  |

*^1^Pooled proportions for multiply imputed data*

There was baseline imbalance between treatment groups in 15 variables prior to PS matching. 6,091/9,244 treated subjects were matched. There were no controls with a PS over about 0.95 so this explains why not all cases could be matched (Figure S3).

Amongst the 6,091 untreated matched subjects, 3,446 were used once in the matching, 1,404 were used twice, 615 were used three times, 333 were used four times and 290 were used five times.

Following PS matching, in the matched sample, a lower proportion of treated individuals had self-harmed in the past with the timing not known (while slightly higher proportions had self-harm histories recorded for the past year and over a year ago).
