# Supplementary material for: Suicide and all-cause mortality following routine hospital management of self-harm: Propensity score analysis using multicentre cohort data
Source: PLoS One. 2018 Sep 27;13(9):e0204670. doi: 10.1371/journal.pone.0204670 (PMC6161837; doi:10.1371/journal.pone.0204670)
Supplement: S7 Table — (DOCX) [file pone.0204670.s007.docx]

**S7 Table:** Outpatient psychiatric referral: Mean and range of propensity score by treatment group

|  | **No. of observations** | **Mean PS** | **Minimum PS** | **Maximum PS** |
| --- | --- | --- | --- | --- |
| Untreated | 6091 | 3385661 | .0463714 | .9316761 |
| Treated | 6091 | .3386643 | .0461436 | .9267116 |
| Total | 12182 | .3386152 | .0461436 | .9316761 |

The frequency density of Figure S3b shows that the distance in propensity score from each treated individual to the matched pair is below 0.01 for most pairs.
