# Supplementary material for: Suicide and all-cause mortality following routine hospital management of self-harm: Propensity score analysis using multicentre cohort data
Source: PLoS One. 2018 Sep 27;13(9):e0204670. doi: 10.1371/journal.pone.0204670 (PMC6161837; doi:10.1371/journal.pone.0204670)
Supplement: S8 Table — (DOCX) [file pone.0204670.s008.docx]

**S8 Table**: Psychiatric inpatient admission: Baseline vs. PS matched covariate balance of PS factors, imputed data (N=31,725^1^)

| Subgroup | Baseline untreated, % | Baseline treated, % | Standardised difference | Matched untreated, % | Matched treated, % | Standardised difference |
| --- | --- | --- | --- | --- | --- | --- |
| Total | 94.3 (29,925) | 5.7 (1,800) |  | 50.0 (1,761) | 50.0 (1,761) |  |
| Male | 41.6 | 48.1 |  | 47.4 | 47.9 |  |
| Female | 58.4 | 51.9 | -0.13 | 52.6 | 52.1 | -0.01 |
|  |  |  |  |  |  |  |
| Age 16 to 24 | 36.3 | 18.1 | -0.42 | 17.8 | 18.5 | 0.02 |
| Age 25 to 44 | 45.1 | 46.3 | 0.03 | 42.6 | 47.0 | 0.09 |
| Age 45 to 64 | 16.1 | 20.7 | 0.12 | 30.2 | 20.8 | -0.22 |
| Age 65+ | 2.6 | 14.9 | 0.44 | 9.4 | 13.6 | 0.13 |
|  |  |  |  |  |  |  |
| Self-poison | 84.2 | 74.1 | -0.25 | 74.4 | 75.1 | 0.02 |
| Self-cut | 11.8 | 14.4 | 0.08 | 15.9 | 14.1 | -0.05 |
| Other self-injury | 4.0 | 11.5 | 0.28 | 9.7 | 10.8 | 0.04 |
|  |  |  |  |  |  |  |
| Any previous psychiatric treatment | 53.4 | 79.2 | 0.57 | 76.9 | 78.8 | 0.04 |
|  |  |  |  |  |  |  |
| Any current psychiatric treatment (including GP) | 40.6 | 72.1 | 0.67 | 57.9 | 72.1 | 0.30 |
|  |  |  |  |  |  |  |
| *Previous self-harm* |  |  |  |  |  |  |
| None | 36.2 | 26.5 | -0.21 | 31.5 | 26.5 | -0.11 |
| In the past year | 30.2 | 45.9 | 0.33 | 37.0 | 45.9 | 0.18 |
| More than 1 year ago | 26.2 | 24.8 | -0.03 | 27.1 | 24.8 | -0.05 |
| Time not known | 7.3 | 2.8 | -0.21 | 4.4 | 2.8 | -0.09 |
|  |  |  |  |  |  |  |
| Alcohol taken | 59.9 | 37.8 | -0.45 | 41.4 | 38.6 | -0.06 |
|  |  |  |  |  |  |  |
| *Problems precipitating self-harm* |  |  |  |  |  |  |
| Relationship with partner | 37.9 | 22.6 | -0.34 | 25.3 | 23.1 | -0.05 |
| Relationship with family | 19.3 | 15.8 | -0.09 | 16.8 | 16.0 | -0.02 |
| Relationship with others | 7.6 | 6.0 | -0.06 | 6.9 | 6.1 | -0.03 |
| Work/study | 13.6 | 11.6 | -0.06 | 11.9 | 11.6 | -0.01 |
| Money | 11.2 | 11.8 | 0.02 | 12.7 | 11.7 | -0.03 |
| Housing | 8.7 | 10.1 | 0.05 | 10.6 | 9.0 | -0.02 |
| Substance misuse | 5.4 | 5.7 | 0.01 | 6.4 | 5.8 | -0.02 |
| Physical health | 7.9 | 11.3 | 0.11 | 11.6 | 11.0 | -0.02 |
| Response to mental health symptoms | 15.4 | 44.8 | 0.67 | 45.1 | 43.7 | -0.03 |
| Bereavement | 7.2 | 8.1 | 0.03 | 8.2 | 8.0 | -0.01 |
| Abuse | 5.0 | 6.1 | 0.05 | 6.3 | 6.1 | -0.01 |
|  |  |  |  |  |  |  |
| Mean IMD score (high = deprived) | 32.0 | 24.7 | -0.37 | 24.4 | 25.0 | -0.04 |
|  |  |  |  |  |  |  |

*^1^Pooled proportions for multiply imputed data*

1,761 of the 1,800 individuals admitted to a psychiatric bed were matched to an untreated pair. Amongst the matched untreated pairs, 1,357 were used once, 286 were used twice, 102 were used three times and 16 were used four times.

Prior to matching, imbalance was present in 11 of the variables. PS matching resulted in balancing all but three of the variables. There remained a higher proportion of individuals aged 65 and over who received psychiatric admission in the matched sample, as well as a higher proportion of untreated individuals aged 45 to 64. Whilst the degree of balance improved following PS matching, there remained a higher proportion of individuals in the matched treated group who were receiving psychiatric treatment at the time of the self-harm and who had self-harmed in the past year.
