# Supplementary material for: Suicide and all-cause mortality following routine hospital management of self-harm: Propensity score analysis using multicentre cohort data
Source: PLoS One. 2018 Sep 27;13(9):e0204670. doi: 10.1371/journal.pone.0204670 (PMC6161837; doi:10.1371/journal.pone.0204670)
Supplement: S9 Table — (DOCX) [file pone.0204670.s009.docx]

**S9 Table:** Psychiatric inpatient admission: Mean and range of propensity score by treatment group

|  | **No. of observations** | **Mean PS** | **Minimum PS** | **Maximum PS** |
| --- | --- | --- | --- | --- |
| Untreated | 1761 | .1467571 | .0026388 | .6868768 |
| Treated | 1761 | .1467602 | .0026407 | .6872246 |
| Total | 3522 | .1467586 | .0026388 | .6872246 |

The frequency density of Figure S4b shows that the distance in propensity score from each treated individual to the matched pair is below 0.005 for most pairs.
