# Supplementary material for: Suicide and all-cause mortality following routine hospital management of self-harm: Propensity score analysis using multicentre cohort data
Source: PLoS One. 2018 Sep 27;13(9):e0204670. doi: 10.1371/journal.pone.0204670 (PMC6161837; doi:10.1371/journal.pone.0204670)
Supplement: S3 Fig — (DOCX) [file pone.0204670.s012.docx]

**S3 Figure**: Outpatient psychiatric referral: Propensity score in treated and untreated (imputed sample, N=29,889)

There was common support for propensity scores up to 0.6 for individuals referred to outpatient mental health care. Above this threshold there were very few untreated individuals, so matching was performed.
