# Supplementary material for: Suicide and all-cause mortality following routine hospital management of self-harm: Propensity score analysis using multicentre cohort data
Source: PLoS One. 2018 Sep 27;13(9):e0204670. doi: 10.1371/journal.pone.0204670 (PMC6161837; doi:10.1371/journal.pone.0204670)
Supplement: S4 Fig — (DOCX) [file pone.0204670.s013.docx]

**S4 Figure**: Outpatient psychiatric referral: Propensity score distance (D) between treated and untreated (imputed sample, N=6,091 untreated subjects)
