# Supplementary material for: Suicide and all-cause mortality following routine hospital management of self-harm: Propensity score analysis using multicentre cohort data
Source: PLoS One. 2018 Sep 27;13(9):e0204670. doi: 10.1371/journal.pone.0204670 (PMC6161837; doi:10.1371/journal.pone.0204670)
Supplement: S5 Fig — (DOCX) [file pone.0204670.s014.docx]

**S5 Figure**: Psychiatric inpatient admission: Propensity score in treated and untreated (imputed sample, N=31,725)

There was common support for propensity scores up to around 0.2 for individuals admitted to a psychiatric bed. Above this threshold there were very few untreated individuals, so matching was performed.
